# The vacated space of volume/price of the drugs centralized procurement with quantity in secondary and above public hospitals of China

**DOI:** 10.1186/s12913-024-11217-3

**Published:** 2024-06-29

**Authors:** Jingliang Wang, Siyu Zhang, Canghong Wang, Jun Li, Rui Wang, Liang Zhu

**Affiliations:** 1 Department of Health Service Management and Medical Education, School of Public Health, Air Force Medical University, Xi’an, 710032 Shaanxi China; 2 The Ministry of Education Key Lab of Hazard Assessment and Control in Special Operational Environment, Xi’an, Shaanxi 710032 China; 3 Xi’an Peihua University, Xi’an, Shaanxi 710125 China; 4 Department of Burns and Cutaneous Surgery, The First Affiliated Hospital of Air Force Medical University, Xi’an, Shaanxi 710032 China; 5 Archives Room, Air Force Medical University, Xi’an, Shaanxi 710032 China

**Keywords:** National Centralized Drug Procurement policy, Difference in difference, Exchange a sales volume for a price

## Abstract

**Background:**

In 2018, the National Centralized Drug Procurement (NCDP) policy has been implemented in 11 provinces, and promoted across the country in 2019. The main feature of the policy is “volume for price”, therefore, it is necessary to measure the price relationship, not only to reduce the price of drugs, reduce the burden of patients' medical costs, but also facilitate pharmaceutical companies to access enough innovation incentives. The aim of this study was to assess the vacated space effect of the drug centralized procurement by national organizations in exchange of price for quantity.

**Methods:**

A difference-in-differences (DID) model was employed to analyze the effect of the 4 + 7 pilot drugs centralized purchasing policy on drug sales volume and selected versus clinically substitutable unselected varieties, using observational data from 2018 to 2019. We compared drug procurement data between secondary and above public hospitals in pilot and non-pilot cities throughout China.

**Results:**

The study showed that the average treatment effect (ATE) of sales in the in-hospital market for the selected supply varieties in centralized purchasing is -0.42, and with a sales volume of 0.49. This indicates a volume-price vacated space of 1.16 ~ 1.17 DDD (defined daily dose)/Yuan, implying that for every 1 defined daily dose (DDD) increase in reported volume, the standardized price decreased by 1.16–1.17 Yuan. The ATE of in-hospital market sales for drugs not selected in centralized procurement shows a decrease of 0.13. This finding highlights the presence of the price linkage effect. The ATE of sales volume is 0.57, indicating a volume-price space of 4.38 ~ 4.39 DDD/Yuan for unselected drugs, approximately 3.75 higher relative to that of the selected ones.

**Conclusions:**

The ratio of the volume-price space of clinically substitutable unselected and selected drugs may serve as direct evidence for evaluating the shift from centralized purchasing of drug varieties to clinically substitutable other ones. To strengthen the volume-based negotiation approach and maximize the effectiveness of centralized purchasing policies, we recommend the strategic implementation of a three-tiered centralized purchasing system, the expansion of drug coverage, and the introduction of relevant constraints and incentives.

## Background

The rising cost of drugs has significantly increased healthcare expenditures worldwide. As a result, governments and academic communities have been investigating ways to lower the cost of pharmaceuticals without sacrificing their quality. The market for pharmaceutical products is imperfectly competitive, necessitating government control [1]. Studies have reported that drug bidding can efficiently control the unreasonable rise of drug prices in many countries, thus acting as a robust and sustainable solution [2–4].

The centralized drug procurement policy of the Chinese government aims to lower drug costs, expedite the creation of a nationally united, open, and centralized drug procurement market, and effectively lessen the burden of drug use on the general public [5]. This policy mandates the National Health Security Administration (NHSA), which arranges competitive bidding and price negotiation based on the overall annually agreed procurement volume, to receive the agreed procurement volume of each drug in the procurement list from each public medical institution. The bid was won by the pharmaceutical producers with the lowest prices [6]. This regulation applies to all public medical facilities, including secondary public hospitals (49%) [7].

Interval economies of scale and marginal effects imply that national centralized drug procurement (NCPD) can affect the amount and cost of drugs [8, 9]. This strategy takes advantage of the negative relationship between capacity and price to boost purchasing power by increasing the procurement size [10–12]. Pharmaceutical corporations consider the economic leverage between market shares and net profit per unit when bidding, leading to lower drug prices. Hospitals then use targeted procurement to optimize their supply networks [11, 13]. The development and inflection point of volume growth can be predicted by examining the volume and price margin and the ratio of the winning bid price to market share, offering key insights and direction for medicine procurement.

Most empirical research uses a before-and-after policy impact comparison analysis on the centralized procurement method. The research mainly uses data from a single pilot city or area for data processing [14]. For example, Wang et al. (2021) conducted a comparative analysis based on drug sales data in the pilot area before and after the implementation of centralized purchasing policies [15]. Chen et al. (2020) also explored the factors driving drug expenditure using order data from the pilot drug procurement survey in Shenzhen, using a three-factor index system analysis [16]. Huang et al. (2019) assessed the price of selected drugs (ten drug categories in China's "4 + 7" purchasing program) in the pilot areas with centralized purchasing and bidding prices in non-pilot areas using the median price ratio (MPR) as an indicator. They also analyzed changes in the standard burden cost of the drugs using the WHO (World Health Organization)/HAI (Health Action International) method. Wang & Yang (2021) evaluated the policy shock effects of the drug price index (DPI) in a month using a single-group breakpoint time series regression analysis [17, 18]. These investigations have produced some empirical data. Nonetheless, only a few studies have examined the volume/price void impacts of centrally procured medications in secondary and higher public hospitals in China.

This study aimed to evaluate the changes in the volume-price connection following the adoption of the centralized purchasing strategy using the drug purchase and sale data of public hospitals in the pilot areas that are above the secondary level based on the DID model. A lot of centralized procurement policy data were obtained in the early stages. Expert consultation and literature review were conducted to establish a set of indicators for outcome and explanatory variables. An empirical model was constructed for the volume and price of space vacated by centralized purchasing. The methods for quantitatively characterizing the amount and price of vacated space were also clarified. The model was divided into three sections: DID model and fixed effects (FEs). The DID model is used to assess the parallel effects of two sub-models. Second, the event control was divided into two categories: drug control (various generic names under the same ATC subclass level vs. selected supply variations under centralized procurement) and regional control (pilot city vs. non-pilot main city). Finally, the model was used to measure the space for volume-price trade-offs of drugs, examine the expectations of medication policy, collect evidence of centralized procurement variety shifts, and suggest tendentious policy recommendations.

## Methods

### Data sources

The data of the procurement volume of pilot drugs were obtained from the reported volume of the pilot regions listed in the Shanghai Sunshine Pharmaceutical Purchasing Network (SSPPN).

The drug purchase and sales data of public hospitals above the secondary level in the centralized procurement in the pilot areas were obtained from the National Hospital Sales Database (NHSD) and the National Sample Hospital Sales Database of Pharnexcloud (NSHSDYR). These data are widely used to make business decisions. The NSHSDYR database contains a variety of reliable data, including cooperative hospital procurement data, cooperative pharmaceutical company delivery data, and cooperative data of some pharmaceutical circulation enterprises. Notably, data are stored in the NHSD database after professional data cleaning and integration. The NHSD data include the number of city-level hospitals, hospital beds, hospital types, regional diseases, local medical level, etc., covering more than 1,400 urban secondary and above public hospitals (beds ≥ 100), spanning 24 provinces and 25 main cities. Drugs include chemical drugs /bio-pharmaceutical drugs and Chinese proprietary (patent) medicines, of which 15 major categories are chemical drugs /bio-pharmaceutical drugs (90 subcategories and 4,800^+^ common varieties). The NHSD database accounts for about 11% of the national hospital drug market. The Health Statistics Annual Report (2019) identified 12,000^+^ medical institutions above the secondary level in the whole country.

### Regional panel data

Annual data for major regional cities were obtained from a study conducted by the National Statistics Bureau of China and published on 21 May 2023 (title: *Annual Data for Major Cities*). The regional statistical indicators included the number of outpatients and emergency treatment visits to healthcare institutions, the expenditure on medicines of the basic medical insurance fund (10,000 *yuan*), and the consumer price index for western medicines (previous year = 100).

### Sample selection and control setting

#### Sample drugs

The packaging specifications, usage, and dosage of the marketed drugs of various manufacturing enterprises are different. The quantity and price of drugs are standardized to make the quantity and price benchmarks comparable according to the DDD of the ATC/DDD system of the WHOCC—ATC/DDD index. The quantity and price of drugs are converted into the price ratio of drugs with DDD as the smallest unit of preparation following the rules of the price bureau for the difference of drugs [19]. The only drugs excluded from following the specifications in centralized procurement include: (1) pemetrexed disodium, flurbiprofen ester, and dexmedetomidine hydrochloride injections because the injectable dosage forms are mainly used in inpatient services; (2) gefitinib and imatinib, which are covered by the tax reduction policy on anticancer drugs affected by the dual policies in procurement volume and price; (3) products with secondary regulations since the main product regulations are determined by the "4 + 7" urban drug centralized procurement document of the Joint Procurement Office (JPO) as the reduced data capacity. Only the commonly prescribed tablets and dispersion used in outpatient and emergency services were used in this study. The minimum data recording unit was "standardized DDD-City-Quarter". A total of 17,664 data records were integrated after excluding digestive and metabolic drugs (no generic drugs for Smectite Powder were found). Finally, the purchase and sales data from NCPD and substitute drugs for other generic drugs under WHO/ATC 4-level used in public hospitals above the secondary level in pilot/non-pilot cities were included in the study. The top 20 drugs commonly prescribed in outpatient and emergency services and some clinically substitutable varieties for other generic drugs were then screened (Table 1). Finally, 93 drug varieties with generic names (5 major categories and 13 ATC 4-level subcategories, including cardiovascular, neurological, systemic anti-infective, blood and haematopoietic, and respiratory drugs) were included in this study.

**Table 1:** Generic names and ATC classification of drugs included in the study

SN	ATC 1	ATC 2	ATC 3	ATC 4	CADN
1	CARDIOVASCULAR SYSTEM	LIPID MODIFYING AGENTS	LIPID MODIFYING AGENTS,PLAIN	HMG CoA reductase inhibitors	Atorvastatin*
					Rosuvastatin*
					Fluvastatin
					Lovastatin
					Pitavastatin
					Pravastatin
					Simvastatin
		AGENTS ACTING ON THE RENIN-ANGIOTENSIN SYSTEM	ANGIOTENSIN II ANTAGONISTS, PLAIN	Angiotensin II antagonists, plain	Azilsartan medoxomil
					Olmesartan medoxomil
					Irbesartan*
					Candesartan
					Losartan*
					Telmisartan
					Valsartan
					Olmesartan medoxomil
			ANGIOTENSIN II ANTAGONISTS, COMBINATIONS	Angiotensin II antagonists and diuretics	Olmesartan medoxomil and diuretics
					Irbesartan and Diuretics*
					Candesartan and diuretics
					Losartan and diuretics
					Telmisartan and diuretics
					Valsartan and diuretics
			ACE INHIBITORS, PLAIN	ACE inhibitors, plain	Benazepril
					Fosinopril*
					Captopril
					Lisinopril*
					Ramipril
					Imidapril
					Perindopril
					Enalapril*
					Enalaprilat
		CALCIUM CHANNEL BLOCKERS	CSELECTIVE CALCIUM CHANNEL BLOCKERS WITH MAINLY VASCULAR EFFECTS	Dihydropyridine derivatives	Azedipine
					Amlodipine*
					Amlodipine and folic acid
					Barnidipine
					Benidipine
					Felodipine
					Lacidipine
					Lercanidipine
					Manidipine
					Nitrendipine
					Cilnidipine
					Nifedipine
					Levamlodipine
2	BLOOD AND BLOOD FORMING ORGANS	ANTITHROMBOTIC AGENTS	ANTITHROMBOTIC AGENTS	Platelet aggregation inhibitors excl. heparin	Acetylsalicylic acid
					Aspirin and dipyridamole
					Beraprost
					Clopidogrel *
					Ticlopidine
					Sarpogrelate
					Dipyridamole
					Ticagrelor
					Cilostazol
					Iloprost
					Indobufen
3	ANTIINFECTIVES FOR SYSTEMIC USE	ANTIVIRALS FOR SYSTEMIC USE	DIRECT ACTING ANTIVIRALS	Nucleoside and nucleotide reverse transcriptase inhibitors	Adefovir dipivoxil
					Tenofovir alafenamide
					Emtricitabine
					Entecavir *
					Lamivudine
					Zidovudine
					Telbivudine
					Tenofovir Disoproxil *
		ANTIBACTERIALS FOR SYSTEMIC USE	OTHER BETA-LACTAM ANTIBACTERIALS	Second-generation cephalosporins	Cefprozil
					Cefuroxime *
					Cefaclor
4	NERVOUS SYSTEM	PSYCHOANALEPTICS	ANTIDEPRESSANTS	Selective serotonin reuptake inhibitors	Escitalopram*
					Vortioxetine
					Fluvoxamine
					fluoxetine
					Paroxetine*
					Sertraline
					Citalopram
		PSYCHOLEPTICS	ANTIPSYCHOTICS	Diazepines, oxazepines, thiazepines and oxepines	Olanzapine*
					Quetiapine
					Clozapine
				Other antipsychotics	Aripiprazole
					Blonanserin
					Risperidone*
					Paliperidone
		ANTIEPILEPTICS	ANTIEPILEPTICS	Other antiepileptics	Compound Benzenamine
					Gabapentin
					Lacosamide
					Lamotrigine
					Pregabalin
					Topiramate
					Asarone
					Vanillin
					Levetiracetam*
					Zonisamide
5	RESPIRATORY SYSTEM	DRUGS FOR OBSTRUCTIVE AIRWAY DISEASES	OTHER SYSTEMIC DRUGS FOR OBSTRUCTIVE AIRWAY DISEASES	Leukotriene receptor antagonists	Pemirolast
					Compound Tranilast
					Montelukast*
					Pranlukast
					Tranilast

(1) "Generic name*" indicates the selected supply varieties; (2) the study only included drug varieties in oral dosage form; (3) the DDD values of the drugs in this study are queried using the official WHO channel (https://www.whocc.no/atc_ddd_index/), with the price conversion following the "Notice of National Development and Reform Commission on the Issuance of the Rules on Drug Differential Price" (NDRC Price [2011] No. 2452); the standard price of the minimum preparation unit is calculated using the DDD value and the conversion factor

#### Sampling regions

The pilot was conducted in 11 cities, including Beijing, Tianjin, Shanghai, Chongqing, Shenyang, Dalian, Xiamen, Guangzhou, Shenzhen, Chengdu, and Xi'an. The NSHSDYR contains data from 25 main cities, such as Beijing, Chengdu, Chongqing, Fuzhou, Guangzhou, Guiyang, Harbin, Hangzhou, Hefei, Hohhot, Jinan, Kunming, Nanjing, Shanghai, Shenzhen, Shenyang, Shijiazhuang, Taiyuan, Tianjin, Urumqi, Wuhan, Xi'an, Changchun, Changsha, and Zhengzhou.

In this study, nine pilot cities, including Beijing, Tianjin, Shanghai, Chongqing, Shenyang, Guangzhou, Shenzhen, Chengdu, and Xi'an, were selected. The control group included 15 major provincial capitals, such as Fuzhou, Guiyang, Harbin, Hangzhou, Hohhot, Jinan, Kunming, Nanjing, Shijiazhuang, Taiyuan, Urumqi, Wuhan, Changchun, Changsha, and Zhengzhou.

The treatment and control groups were set up as pilot/non-pilot regions, totaling 24 major cities with city ID codes (Table 2). The grouping was based on whether the policy has implemented centralized drug procurement, taking into account the coverage of the database. The quarterly time unit of 2018 and 2019 were selected as the observation time window for the natural experiments to avoid the impacts of complex factors, such as the centralized purchasing expansion and the multiple policies of the health insurance drug price negotiation, and the prevention and control of the COVID-19. The study was based on the 1st quarter of 2019 since the "4 + 7" NCPD ended (or started) its implementation of the selection results at the end of March 2019. The minimum data unit was "Generic names-City-Quarter", including 55,840 data samples.

**Table 2:** The ID codes of the 24 major cities

Cities	Beijing	Tianjin	Shanghai	Chongqing	Shenyang	Guangzhou	Shenzhen	Chengdu	Xi'an	Fuzhou	Guiyang	Harbin
ID	1	2	3	4	5	6	7	8	9	10	11	12
Cities	Hangzhou	Hohhot	Jinan	Kunming	Nanjing	Shijiazhuang	Taiyuan	Urumqi	Wuhan	Changchun	Changsha	Zhengzhou
ID	13	14	15	16	17	18	19	20	21	22	23	24

City IDs 1 to 9 are the pilot treatment group and the rest are the non-pilot control group

### Empirical modelling

#### Vacated space effect

The double difference method was used to compare the changes in the standardized prices of centralized procurement varieties, similar alternative non-centralized procurement varieties, and the changes in sales volume before and after the implementation of the centralized procurement policy. The space of quantity and price replacement were then quantitatively described. Furthermore, the size of the price replacement space was quantitatively described based on the ratio of the average increment effect of clinical drug use in public hospitals above the secondary level in the pilot areas to the average price reduction effect under the blank control of centralized procurement drugs.

1 $${\text{SPACE}}_{\text{V}/\text{P}}\text{=}\frac{{\upbeta }_{\text{V}}}{{\upbeta }_{\textrm{P}}}$$

βv and βp represent the average processing effect of sales and the average processing effect of sales, respectively.

#### Variable description

Table 3 the definitions and descriptions of the model covariates.

**Table 3:** Description of model variables and proxies

Variable Type	Variable Name	Variable Generation	Indicator Description and Assignment Definition
Outcome Variables	Standardized sales	*Y*_*p*_	Quarterly sales of drugs of the same generic name in major cities after standardization
	Standardized sales volume	*Y*_*n*_	Quarterly sales volume of drugs of the same generic name in major cities after standardization
	Standardized reporting volume	*Y*_*n0*_	Standardized reporting volume of public announcements on the Sunshine Purchasing Network in pilot regions
Core explanatory variables	Control variables	*Treat*_*m*_	Treatm Pilot = 1; Non-pilot = 0
	Event variable	*Post*_*t*_	Post-intervention = 1; Pre-intervention = 0
Moderating variables	City Virtual Variables	*m*	M-1 Virtual variables to describe city FEs, M = 24
	Common Name Virtual Variables	*i*	I-1 Virtual variables to describe drug FEs, I = 20
	Time Virtual Variables	*t*	The study uses panel data from 2014–2018 (T = 5) with a cross-section of 24 cities and T-1 Virtual variables describing year FEs
	Quarter Virtual Variables	*q*	Q1 2018—Q4 2019 q = -4, -3, -2, -1, 1, 2, 3
Other explanatory variables	Number of outpatient consultations	*X*_*treat*_	Annual number of outpatient and emergency services visits to medical institutions in major cities (billions of visits)
	Per capita expenditure on medicines under medical insurance	*X*_*outcome*_	Per capita expenditure on medicines covered by basic medical insurance (yuan)
	Consumer price index for western medicines	*X*_*CPI*_	Retail price index of urban commodities in the western medicine category (previous year = 100)

(1) in order to avoid the influence of heteroscedasticity, the natural logarithm of the original data set was used; (2) the standardization of the outcome variable "volume" refers to the calculation of the minimum formulation unit (DDD) as the basic unit of multiplication; (3) the standardization of "price" is the volume multiplied by the spread coefficient to convert to the standardized price

#### Fixed Effects (FEs)

It was assumed that there were no omitted variables associated with the explanatory variables in the research model setting. However, this is a strong assumption, particularly in natural experiments where there may be limited data capacity in the database and unobservable environmental variables, as well as indescribable individual heterogeneity. Notably, individual differences do not fluctuate over time (time-invariant) if the large environmental variables do not vary with individuals (individual invariant). Therefore, a panel data set can be used. In this study, panel data FEs were introduced to compensate for the omitted variable bias. The TWFE model was also introduced, expressed as follows:

2 $${\text{Y}}_{\rm{it}}\text{=}{\rm{x}}_{\text{it}}{\beta+}{\rm{z}}_{\text{i}}{\delta+}\lambda_{\rm{t}}\text{+}\mu_{\rm{i}}\text{+}\epsilon_{\rm{it}}$$

*Y*_*it,*_* x*_*it,*_* λ*_*t,*_* μ*_*i,*_ and *ε*_*it*_ represent outcome variables, control variables (other explanatory variables), time FEs, individual FEs, and time individual random disturbance error terms, respectively. The significance of the panel data FEs was examined through tests to consider whether to include them in the DID model.

#### Difference-in-Differences (DID)

DID describes the average treatment effect (ATE) of a policy intervention on the treatment group via the construction of interaction identification based on a counterfactual framework that assesses the changes in the outcome variable in the states where the policy intervention occurs or does not occur [20, 21]. In this study, the control group represents the counterfactual state. The changes in the control group were approximated as the changes in the treatment group with no policy intervention during the trial period. The first-order difference was subtracted between the treatment and control groups before and after the trial to obtain the DID effect from the double treatment as the intervention effect of the policy. The econometric method of implementation was used to analyze the policy effects of the NCPD. Specifically, the results of the counterfactual change by varying the outcomes from the control group being free of the intervention, thus making the treatment group hypothetically free of the intervention. The model was set up as follows:

3 $${\mathrm Y}_{\mathrm{kimt}}\;=\alpha\;+\;\beta\;\left({\mathrm{Treat}}_{\mathrm m}\;\times\;{\mathrm{Post}}_{\mathrm t}\right)\;+\;\lambda_{\mathrm i}\;{\mathrm{CADN}}_i\;+\;\mu_{\mathrm m}\;{\mathrm{City}}_{\mathrm m}\;+\;\gamma_{\mathrm t}{\mathrm T}_{\mathrm t}\;+\;\varepsilon_{\mathrm{kimt}}$$

*k*, *i*,* m*, and* t* represent the smallest formulation unit (DDD), generic name of the drug, main city, and time (years), respectively. The outcome variables,${\text{Y}}_{\text{kimt}}$ represent the post-labeling sales price/sales volume while *Treat*_*m*_ represents pilot identification variables. *Treat*_*m*_ = 1 and *Treat*_*m*_ = 0 represent the treatment and control groups, respectively. ${\text{Post}}_{\text{t}}$ represents the policy intervention identifying variable, the point of implementation of centralized purchasing (Q2 2019 and beyond (9 pilot cities ended their implementation at the end of March among 20 supply varieties under study, *Post*_*t*_ = 1). The interaction coefficient of the identifying variable *β* indicates the policy intervention effect. *λ*_*i,*_* μ*_*m,*_* γ*_*t,*_ and *ε*_*kimt*_ represent drug generic FEs, city FEs, time FEs, *and* the random error term, respectively.

#### Parallel trend

Deviations may occur in the purchase and sales volume and price effects of centralized purchasing if the empirical study fails to control its endogenous selection bias in the sample. In this study, the sample selection was considered as exogenous selection released from the market demand after the centralized purchasing drug varieties were submitted by the region, approved by the JPO, and determined by consulting experts. However, it is unclear whether the selection of pilot cities is entirely chosen by the exogenous policies. This is supported by the data on the sales of drugs in the sample hospitals of main cities. The sales volume and sales of drugs supplied by centralized purchasing in the pilot cities were significantly higher than those supplied by non-pilot main cities before the implementation of centralized purchasing (*P* < *0.05*).

However, the effect of the bargaining space may be exaggerated in centralized purchasing if the effects of subjective deployment of policy choices on the centralized purchasing results is ignored. The arrival of the inflection point can be expedited where volume growth and price increase would be greatly released if this fallacy is adopted to guide the subsequent purchasing decisions of volume and price, resulting in the overestimation of the market's digestive capacity and the expansion of the pharmaceutical industry. The selection deviations were caused by different initial conditions and time trends in the treatment group (pilot) and the control group (non-pilot). The endogenous selection can be avoided if the pilot selection is random [22]. However, the observed data is a natural experiment exposed to the experimental policy environment under non-controlled factors.

Herein, DID was used to measure the differences between the incremental increase of the treatment and control groups under the centralized purchasing policy in the natural experiment using data from natural observations (matching method in the sense of differences). DID is widely applied to identify the exact cause and effect of the policy intervention based on the premise that the treatment and control groups pass the test of a co-parallel trend (the treatment group is in the same state of time-varying trend as the control group in the absence of the policy intervention; the counterfactual hypothesis) [23]. Although the trend is consistent, the hypothesis on the parallel trend test cannot be directly tested because the counterfactual results cannot be directly observed after the intervention time point [24]. The time co-trend can be indirectly tested through the pre-event trend test [22]. Furthermore, the endogenous compositional changes can be verified for the differences among cities existing in the centralized procurement before and after the policy intervention if the parallel pre-event trend is consistent with the post-time trend, and the difference matching avoids the robustness test of the endogenous problem hypothesis [20]. In this study, pre-event parallel trend tests were conducted by adding dummy variables for each quarter of the experimental observation period with treatment group interaction terms formulas follows:

4 $${\mathrm Y}_{\mathrm{kimt}}\;\mathrm\alpha\;+\;{\sum_{\mathrm q=-4}^3}\;{\mathrm\beta}_{\mathrm q}\;\left({\mathrm{Treat}}_{\mathrm m}\;\times\;\mathrm{Post}_{\mathrm t}^{\mathrm q}\right)\;+\;{\mathrm\lambda}_{\mathrm i}\;{\mathrm{CADN}}_{\mathrm i}\;+\;{\mathrm\mu}_{\mathrm m}\;{\mathrm{City}}_{\mathrm m}\;+\;{\mathrm\gamma}_{\mathrm t}{\mathrm T}_{\mathrm t}\;+\;{\mathrm\varepsilon}_{\mathrm{kimt}}$$

*q* represents a quarterly dummy variable, Q1 in 2018—Q4 in 2019, *q* = -4, -3, -2, -1, 1, 2, 3, 4. An indirect test was conducted to test whether the common trends are parallel by testing if the interaction coefficients *βq* of the pre-event (*q* = -4, -3, -2, -1) policy interventions are significantly non-zero.

## Results

### Fixed effects test

This model used a panel data set of 24 major cities (collected from 2014 to 2018) to remove the biased estimation results due to omitted variables. The cross-sectional data included the number of outpatient and emergency treatment visits to medical and health institutions in major cities (10,000 plus visits), the expenditures on medicines of the basic medical insurance fund (10,000 *yuan*), and the consumer price index for Western medicines (previous year = 100). The volume of medicines sold by public hospitals above the secondary level of the city was used as the outcome variable to test the bidirectional FEs of the main model (DID) and to perform parameter estimation.

### Descriptive analysis of the panel data set

Stata/MP 17.0 was used to test the data set as balanced panel data (STRONGLY BALANCED) within cities n = 24, for time T = 5, and with a minimum observing data unit as the city-time. Short panel data were used as the data set. The statistical characteristics of the panel data set variables are described in Table 4.

**Table 4:** Descriptive statistics of panel data for 24 cities, 2014–2018

Variables		Mean value	Standard Error	Minimum value	Maximum value	Observation Sample
${\text{Y}}_{\text{it}}$	Overall	6.737339	1.005881	3.993192	8.808556	*N* = 120
	Intergroup		.9761299	4.381501	8.572344	*N* = 24
	Intra-group		.3016524	5.516039	7.681735	T = 5
*X*_*treat*_	Overall	10.05996	.647408	9.13777	11.32176	*N* = 120
	Intergroup		.6570964	9.163018	11.27942	*N* = 24
	Intra-group		.0433003	9.953104	10.20036	T = 5
*X*_*outcome*_	Overall	15.14521	.6662472	13.75068	16.48322	*N* = 120
	Intergroup		.5871281	14.203	16.19157	*N* = 24
	Intra-group		.3327959	14.50997	16.00705	T = 5
*X*_*CPI*_	Overall	4.612283	.0189616	4.546481	4.653008	*N* = 120
	Intergroup		.0137991	4.57707	4.6284417	*N* = 24
	Intra-group		.0132487	4.578677	4.654286	T = 5

(1) To avoid the effect of heteroscedasticity, the natural logarithm of the original data set is taken; (2) the variable description is divided into 3 parts: "Overall", "Between Groups" denotes the longitudinal data set, with the city variable Between Groups = 0, and "Within Groups" denotes the cross-sectional data set, with the time variable Within Groups = 0

**Fig. 1:**
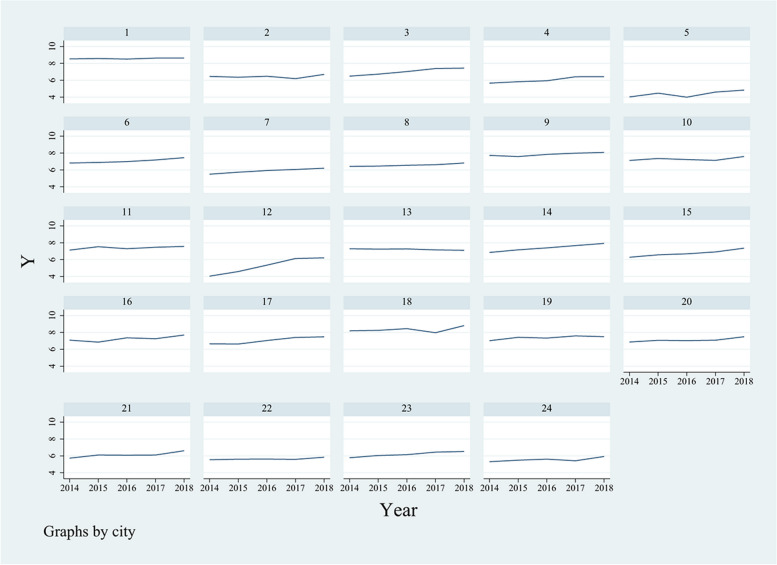
Trends in annual drug sales in sample hospitals in 24 cities, 2014–2018

The time trend of drug sales in public hospitals above the secondary level in 24 major cities is shown in Fig. 1.

Although the annual time trend remained flat or showed an upward trend, the drug sales volume varied in public hospitals above the second level in 25 major cities. Therefore, the pre-event trends in the control and treatment groups should be evaluated to predict endogenous selection differences that do not include the differences in intercity time trends in drug sales volumes to achieve a fully exogenous policy (the NCPD in bulk pilot selection randomization).

### Two-way fixed effects (TWFE) test

A fixed effects (FEs) model was introduced to solve the problem of omitted variables. The individual and time effects were considered in the FE model (TWFE model).

5 $${\text{Y}}_{\rm{it}}\text{=}{\rm{x}}_{\text{it}}{\beta+}{\rm{z}}_{\text{i}}{\delta+}\lambda_{\rm{t}}\text{+}{\mu}_{\rm{i}}\text{+}{\epsilon}_{\rm{it}}$$

The perturbation term consists of ($\lambda_\text{t}\text{+}\mu_\text{i}{{+ \epsilon}}_\text{it}$) composite perturbations. The unobservable variable *μi* represents the intercept term for individual heterogeneity in the panel cross-section. This term is interpreted as an individual FEs. *λt* represents the intercept term for the *t*^*th*^ period of the panel data, and this is interpreted as a time FEs. *Εit* represents a perturbation term that varies with time and individual. The Eq. 2–1 can be estimated using the Least Square Dummy Variable (LSDV) model. Each individual and period can be defined as a dummy variable to regress out the TWEF. Although the different disturbance terms are independent of each other due to the characteristics of panel data, auto-correlation usually occurs between them. The ordinary standard error calculation method assumes that the disturbance terms are independently and identically distributed, indicating that this calculation may be inaccurate. Therefore, the standard error should be estimated using the Cluster-Robust Standard Error (CRSE). The cluster is composed of all observations of each individual at different times [25]. Herein, the observations were allowed to be correlated in the same cluster (city/time). The regression results of the TWEFs model test are shown in Table 5.

**Table 5:** TWEF model test regression

Individual FEs		Time FEs	
Variable	Y_it_	Variable	Y_it_
*X*_*treat*_	-0.168		-0.978
	(0.486)		(0.550)
*X*_*outcome*_	0.641^***^		0.405^**^
	(0.108)		(0.109)
*X*_*CPI*_	2.125		0.335
	(1.761)		(2.175)
Beijing	0	2014 year	0
	(.)		(.)
Tianjin	-1.529^***^	2015 year	0.123^*^
	(0.371)		(0.0543)
Shanghai	-1.106^***^	2016 year	0.209^*^
	(0.279)		(0.0821)
Chongqing	-1.836^***^	2017 year	0.250
	(0.359)		(0.146)
Shenyang	-3.417^***^	2018 year	0.399^*^
	(0.459)		(0.173)
Guangzhou	-1.164^***^		
	(0.146)		
Shenzhen	-1.810^***^		
	(0.476)		
Chengdu	-1.503^***^		
	(0.393)		
Xi’an	-0.486^***^		
	(0.0715)		
Fuzhou	-1.152^*^		
	(0.441)		
Guiyang	-0.983^*^		
	(0.447)		
Harbin	-2.570^***^		
	(0.153)		
Hangzhou	-0.686^***^		
	(0.131)		
Hohhot	-1.087^*^		
	(0.487)		
Jinan	-1.257^**^		
	(0.429)		
Kunming	-0.787^**^		
	(0.216)		
Nanjing	-1.017^***^		
	(0.111)		
Shijiazhuang	-0.203		
	(0.621)		
Taiyuan	-0.705^**^		
	(0.246)		
Urumqi	-1.127^**^		
	(0.342)		
Wuhan	-1.448^***^		
	(0.333)		
Changchun	-2.213^***^		
	(0.141)		
Changsha	-1.605^***^		
	(0.200)		
Zhengzhou	-2.353^***^		
	(0.439)		
Constant term	-9.748		8.705
	(8.128)		(12.33)
Number of observations	120		120
*R*^*2*^	0.959		0.580
*R*^*2*^adjusted value	0.948		0.554

(1) To circumvent the effect of heteroscedasticity, the natural logarithm is taken for the original data set; (2) * *p* < 0.05, ** *p* < 0.01, *** *p* < 0.001

The regressions were conducted separately for individual and time FEs. Beijing was used as the benchmark in the individual FEs regression. The *p*-value of the *F*-test for the original hypothesis "*H*_*0*_*: all u*_*i*_ = *0*" was 0.000, and thus the original hypothesis was rejected (it is assumed that the city FEs (intercept term) exist). The original hypothesis showed that "all individual virtual variables are 0", and thus was rejected. The individual effect existed since the output results showed that most of the virtual variables in the city were significant (*p* < 0.05). The year 2014 was taken as the base period in the regression of the time FEs. The signs of the time effect were positive, and the results were statistically significant for the years of 2015, 2016 and 2018, while the results were not significant for the years of 2014 and 2017. The joint significance of the time dummy variable was assessed at *P* > *F* = *0.0283* < *0.05*. The test rejected the original hypothesis that "there is no time effect", and validated that the model should include time FEs. The TWFEs test showed that the main model DID should include a FEs dummy variable model to address the omitted variable bias.

### Parallel trend test

The parallel trend test was based on the results of the pre-event trend test [26]. The study assumed that the post trends were parallel if the pre-event trends of the treatment and control groups were parallel [20]. The pre-event trend test can be intuitively understood as the interpreted variables of the *t*^*th*^ period before the policy intervention. The differences between the standardized sales and sales volume in the period t before policy intervention were relative to the differences between the two in the base period (quarter before the implementation of the centralized purchasing selection; the first quarter of 2019). The pre-event parallel trend showed that there was no significant change in the differences between the groups of each staged treatment group and the control group before policy intervention. Further analysis can test whether the interaction coefficient of the pre-event policy effect is significantly different from zero and indirectly test whether the pre-event parallel trend holds.

**Fig. 2:**
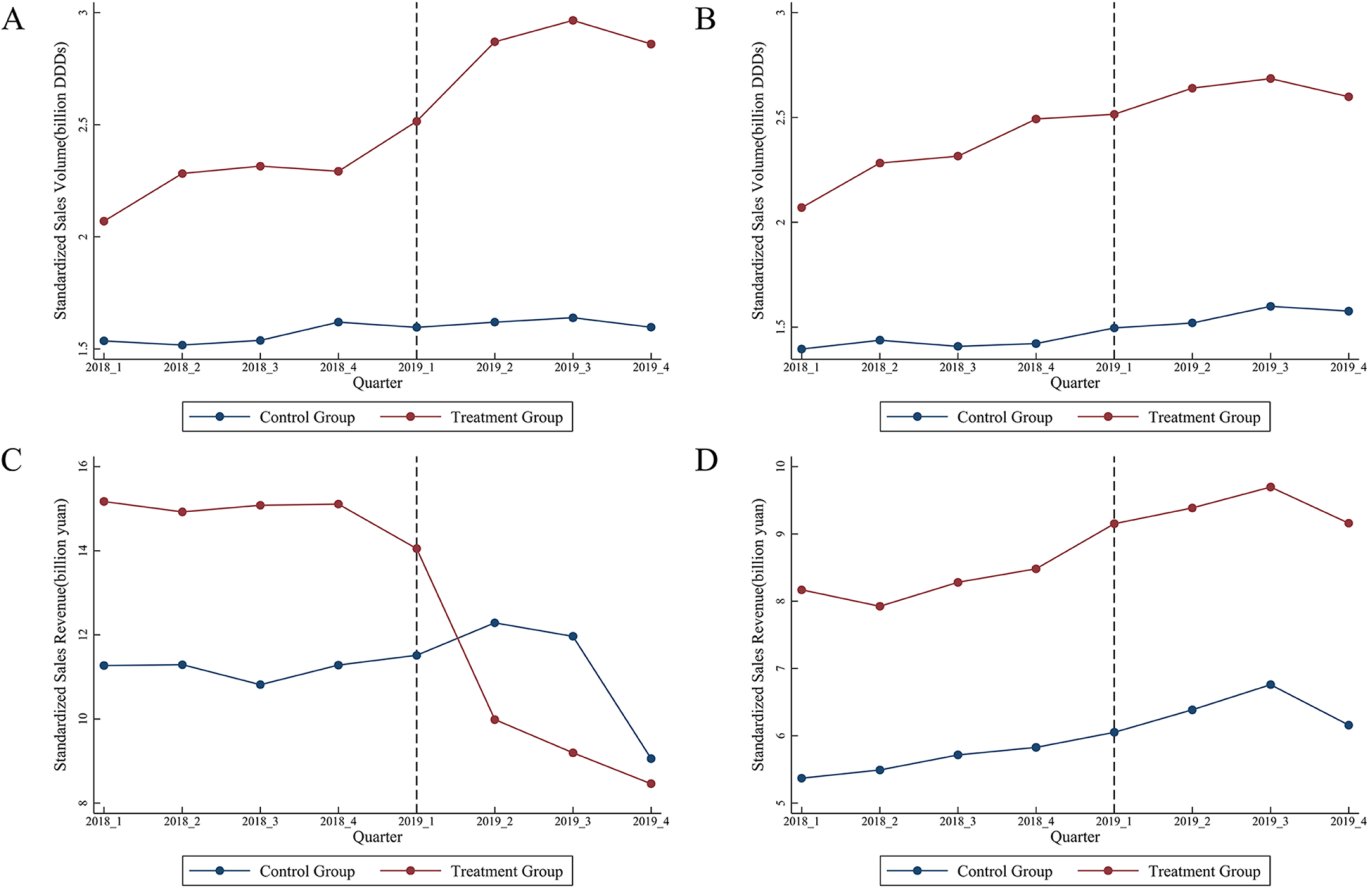
Time trend of standardized sales volume/sale value of selected/non-selected varieties for centralized purchasing

The time trend of sales volume/sales of supply varieties standardized in centralized/non-centralized procurement is shown in Fig. 2. Preliminary results concluded that the time trend was consistent with the changes in the treatment and the control groups in the standardized sales and sales volume of drugs before the implementation of the pilot procurement in the base period. The sales volume significantly increased in the pilot cities in their centralized procurement of selected supply varieties, and sales significantly decreased after the implementation of pilot procurement. Similarly, the sales volume/sales of alternative non-selected varieties followed the same trend. This indicates that the price of unsuccessful drug prices may be gradually affected by the policy shocks and the price spillover effect of the selected drug prices. These changes may be influenced by the increase in inertia-hospital market volume of transactions, which can then decrease due to the impact of the drug health insurance price adjustment.

**Fig. 3:**
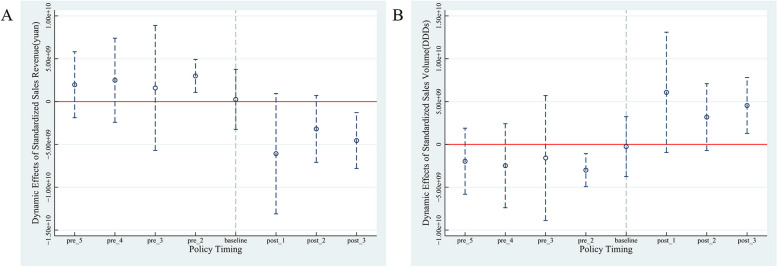
Dynamic effects of in-hospital drug standardized sales/volume sold. Note: The left panel shows the dynamic effects of in-hospital standardized sales (yuan) policy; the right panel shows the dynamic effects of in-hospital standardized sales volume (DDDs) policy

The dynamic effects of the policy intervention were then examined using event analysis to test whether the pre-event effect coefficients were significantly different from the null hypothesis (Fig. 3).

The dynamic effects plot showed that the effect coefficients of in-hospital standardized sales and sales volume in the pre-intervention period (Quarter 1, 2019), with each quarter as a period, for the pre-event (5) periods were mostly concentrated around zero and insignificant at the 95% confidence level. The regression results of the pre-event coefficient test are shown in Table 6.

**Table 6:** Regression of effect coefficients for standardized sales/volume of in-hospital drug sales

Virtual variable	Regression(1)	Regression(2)
	Y_p_	Y_n_
*pre_5*	1.96473e + 09^*^	-3.13985e + 09^*^
	(1.57534e + 09)	(1.00874e + 09)
*pre_4*	2.48973e + 09	-3.49061e + 09^*^
	(2.00528e + 09)	(1.01040e + 09)
*pre_3*	1.58981e + 09	-2.08878e + 09
	(2.98058e + 09)	(1.10854e + 09)
*pre_2*	3.00000e + 09^**^	-3.58695e + 09^**^
	(787,100,016.1)	(602,630,363.6)
*baseline*	257,373,054.7^***^	-7,373,054.7^**^
	(1.43024e + 09)	(730,283,025.8)
Number of observations	17,664	17,664

Regression (1) is in-hospital standardized sales ($); Regression (2) is in-hospital standardized sales (DDDs); (ii) "baseline" is the base period, and "pre_2-5" is Q4 2018—Q1 2018; (iii) * *p* < 0.05, ** *p* < 0.01, *** *p* < 0.001

Regression of the periods found that the original hypothesis (the effect coefficients are indifferent from zero for all periods pre-event) can be rejected, but the regression coefficients for the prior three periods were insignificant. Therefore, the joint test for pre-event period significance *p* = 0.0423 < 0.05 can be parallel to the pre-event trend.

### Differences-in-Differences (DID)

A comparison between selected supply varieties (20 generic names) and unselected varieties (74 generic names) was conducted in centralized purchasing before and after the policy intervention in the 24 major cities. Regression analysis results, including the ATE of the policy intervention, are presented in Table 7.

**Table 7:** Regression of the effect coefficient of in-hospital standardized sales/volume of sales of drugs of selected supply varieties for centralized procurement

	Regression (1)				Regression (2)			
	Effect coefficient	Robust standard error	*t*	*P* >|*t*|	Effect coefficient	Robust standard error	*t*	*P* >|*t*|
Difference (1)	-0.42	0.14	-2.98	0.025	0.49	0.09	3.11	0.021
Difference (2)	-0.13	0.12	-3.45	0.014	0.57	0.06	3.81	0.009

(i) Regression (1) is in-hospital standardized sales ($); regression (2) is in-hospital standardized sales (DDDs); (ii) Difference (1) is the selected supply species; difference (2) is the unsuccessful supply species; and (iii) the study takes the natural logarithm

Empirical evidence showed that the ATE of the centralized purchasing selected supply varieties in-hospital market sales was -0.42, suggesting a decrease of 42% in expenditures on common outpatient and emergency medicines in hospitals. Similarly, the ATE for sales volume was 0.49, indicating a rise of 49% in related medication volume. The volume and price exchange space was found to be 1.16 ~ 1.17 DDD/yuan, implying that for every increase of centralized purchasing reported volume by 1 DDD, the standardized price falls by 1.16 ~ 1.17 yuan.

The ATE of unselected varieties of in-hospital market sales was -0.13, where price linkage effect is beginning to appear. The ATE of sales volume was 0.57. The volume of unselected drugs was measured in the price space of 4.38 ~ 4.39 DDD/Yuan, about 3.75 times as much as the selected ones. The selected supply drugs are commonly used in clinical practice, with high quality and low price. Although the price reduction was evident after centralized procurement, the absolute price reduction space was smaller than that of other drugs in the same category. Therefore, the price reduction in the centralized procurement scale improves the clinical use of the selected medicines, resulting in an increase in the volume and decrease in the price. The prices of other clinically substitute medicines in the same ATC subclasses dropped under the price linkage mechanism, thus decreasing the price and increasing the volume.

These findings provide direct evidence for evaluating the shift of centralized purchasing drug varieties to other clinically substitute drug varieties of the same ATC subclass, indicating that currently available medications do not meet in-hospital demand, and price linkage effects are minimal. Therefore, incorporating the predicted volume and price changes of clinically substitutable drugs, both selected and unselected for centralized procurement, can inform policy decisions regarding the expansion of such programs.

## Discussion

### Preliminary analysis of the inflection point of the released volume growth market

Through the analysis of the dynamic effect of policy intervention in pilot cities, the results indicated that in the 2nd quarter of 2019—3rd quarter in the pilot cities after the full rollout, the effect coefficient of centralized purchasing sales degree intervention was decreased.However, this trend was not observed in non-pilot cities during the same period.

Therefore, this study provides preliminary evidence that the centralized purchasing of medicines at a given stage may have unintended effects on policy-making in the future. In particular, there will be a shift in industry inflection point of volume growth, in which the price reduction effect coefficient will change from negative value to zero to positive. Considering the short observation time and limited sample data available, it is not feasible to determine the centralized purchasing of drugs volume growth generated by the structural effects of the preliminary judgement.

This may be explained by the excess release of patients' in-hospital demand. In this study of 20 centralized purchasing selected supply varieties, there is no increased in-hospital demand for the use of the backlog of concentrated release, but we believe that the demand outbreak of the time nodes is most likely to be observed in the selected varieties of dynamic adjustments. For example, as a class of drugs in the centralized purchasing range for a long time, if policy is not enforced to the extent of the expectations of drug use, it will be excluded from the procurement plan. However, due to previous hoarding of drugs by individual bidding enterprises, stockpiling of drugs by successful enterprises, production line adjustments, and delayed development of disease epidemics, there is a risk of potential explosive release of demand at a given procurement period. This will result in an increase in the quantity of submitted drugs and drug prices in the subsequent procurement cycle.

### Expand the coverage of centralized drug procurement

Centralized drug procurement focuses on two key aspects of coverage: firstly, it ensures the inclusion of alternative medications within the same therapeutic category [27]. Secondly, it considers the variety of clinically useful drugs within a broader category, avoiding overlap and duplication. Focusing on the use of drugs to replace the volume-price relationship, we provide strong evidence for the centralized procurement of selected supply varieties to the same ATC small class of clinically substitutable drugs transfer. That is, the unselected drugs have a higher price volume of the exchange of space compared to the selected drugs. To expand centralized purchasing, it should be included in a pilot in the supply of drug varieties based on the other drugs of the same class which still need to undergo screening bidding and the value of the market. Building upon the drug varieties identified in the pilot project, centralized procurement should be expanded strategically. This expansion should consider two key factors: clinical use patterns and hospital pricing data.By addressing these factors, the program can alleviate patient access issues for "difficult-to-use drugs" while promoting the use of high-quality, cost-effective generic drugs. This focus on generics will ultimately contribute to reducing overall drug costs. By the end of December 2023, the state had successfully conducted nine rounds of collective drug procurement, encompassing 374 medications and achieving an average price reduction exceeding 50%. Notably, the first eight rounds focused on high-value medical consumables, such as heart stents and artificial joints, and secured an impressive average price reduction of over 80% for these items. Combined procurement efforts by local alliances have collectively saved an estimated 500 billion yuan. This significant reduction in pharmaceutical costs has greatly lessened the financial burden on patients and addressed the public's concern about high medical treatment expenses.

### The spillover effect of volume for price on pharmaceutical enterprises and other medical institutions

A large market share can lead to a siphoning effect for pharmaceutical companies, hindering competition and potentially reducing innovation. Even if the original drugs and generic drugs pool the level of fair competition offer, some big pharmaceutical manufacturers still leverage their own market strategies to gain access to the data of the hospital purchases and sales, thereby rapidly modify their prices, alter their bidding strategies to stabilize the original market, reclaim the new issue of the "good land" of the expected results. In the long run, small businesses will be left with narrow centralized purchasing residual market, in the market price linkage mechanism under the coercion of the "thin profit and micro-sales". Consequently, smaller companies struggle to compete for resources needed for research and development. Consequently, the market may become dominated by larger players with a greater capacity for clinical trials and new drug development. This dominance could potentially lead to manipulation for profit maximization. This trend also suggests a growing concentration within the pharmaceutical industry as a result of the drug collection policy.

Regarding other medical institutions, some private hospitals do not participate in the volume purchase because they can obtain the same or even lower purchase price using the public information of the collection price, and then increase the price and sell to increase the income. On the other hand, some private hospital participate in the volume purchase, however, they do not increase their income, leading to losses [28].For private hospitals not participating in volume purchasing, the government can establish a more rigorous monitoring mechanism to ensure transparency of drug pricing. This may include mandating hospitals to publicly disclose their drug procurement costs and selling prices, thereby reducing opportunities for drug price gouging. For private hospitals participating in volume purchasing but experiencing financial losses, the government can offer financial incentives or subsidies to encourage their continued participation. Additionally, hospitals can explore strategies to enhance efficiency and reduce operational costs through improved management practices, such as implementing lean healthcare processes and optimizing supply chain management.

Concerning other types of drugs, a significant reduction was detected for the DDDc of non-winning original drugs. For example, many non-winning original products reduced their price under the “4 + 7” policy [29]. By establishing a volume–price linkage and enhancing competition, the “4 + 7” policy may contribute to shaping of the market mechanism of drug pricing [30]. However, total drug expenditures are not effectively controlled due to the increased use of alternative drugs [31]. To ensure wider access to these benefits, there is a need to optimize drug collection protocols and increase the representation of covered medications within the drug catalogs of major public hospitals.

## Conclusions

The present results demonstrate that the ATE of in-hospital market sales of selected supply varieties under centralized procurement is -0.42, and the ATE of sales volume is 0.49. with a marking volume-price exchange space ranging from 1.16 to 1.17 DDD/yuan. This implies that for each increase of 1 DDD in centralized procurement reported volume, the standardized price decreases by 1.16 ~ 1.17 yuan. For unselected varieties in centralized procurement, the ATE of in-hospital market sales is -0.13, and the ATE of sales volume is 0.57, suggesting that the volume and price space of unselected drugs is approximately 4.38 ~ 4.39 DDD/yuan, about 3.75 higher relative to that of the selected drugs. The price linkage mechanism provides a unique advantage: it simultaneously lowers costs for clinically equivalent drugs (within the same ATC subcategory) while boosting their sales volume. This dynamic within a category, analyzing both volume and price changes (volume-to-price space) for selected and unselected drugs, offers valuable insights for market research and analysis. A ratio > 1 signifies the need to broaden the scope of similar drugs with potential room for downward adjustment of the price in the same category of drugs. Conversely, a ratio ≤ 1 suggests that the respective generic medicines are ideal substitutes for satisfying the original market demand, and further price reductions may not be feasible.

The volume-for-price approach can trigger a spillover effect that impacts pharmaceutical companies and other medical institutions. As a result, these entities should adjust their strategies to address this potential consequence.

### Limitation

The empirical strategy has some limitations in terms of data accessibility and practicality. The limitations are classified as follows: (1) given that the major cities data takes the year as the time span, the year is the minimum time unit of the panel data of 24 main cities from 2014–2018 in the study, while the time unit of the NCPD is the quarter, so there is a bias in the FEs coefficients estimated by the TWFE model; (2) we did not explore the impact of inter-provincial alliances and provincial centralized purchasing on the volume-price relationship, which may create a bias of overestimating the price linkage ripple effect of the NCPD; (3) the study uses drug sales data from public hospitals above the second level in the main cities of the NSHSDYR, there is, therefore, certain degree of structural effect bias; (4) he results may not be directly applicable to centralized purchasing programs for other major drug and medical consumable categories.

To address these limitations, the following improvement measures are proposed: (1) data should be collected from to local annual reports, unified time span, and experts should be invited to describe the other explanatory variables with greater validity, therefore, improve the suitability of TWFE; (2) the volume-price relationship of centralized purchasing at different levels should be evaluated extensively to facilitate the analysis of its policy effects in a hierarchical manner; (3) there should be stratification based on the hospital level in the analysis of volume and price of drug purchases and sales. The analysis should be expanded to the overall in-hospital market. Moreover, the hospital level needs to be accounted as an instrumental variable to correct for the omitted variable bias in the reimbursement of expenditures by health insurance.

## Data Availability

The data sets used and/or analyzed during the current study available from the corresponding author on reasonable request.

## Abbreviations

NCPD: National centralized procurement of drugs
SSPPN: Shanghai Sunshine Pharmaceutical Purchasing Network
NHSD: National Hospital Sales Database
NSHSDYR: National Sample Hospital Sales Database of Yao Rongyun
JPO: Joint Procurement Office
WHO: World Health Organization
HAI: Health Action International
DDD: Defined daily dose
ATC: Anatomical Therapeutic Chemical
DPI: Drug price index
MPR: Median price ratio
DID: Difference-in-differences
ATE: Average treatment effect
Fes: Fixed effects
TWFE: Two-way Fixed Effects
CRSE: Cluster-Robust Standard Error
